# Serum Phenylacetylglutamine Is a Potential Risk Factor for Aortic Stiffness in Patients with Chronic Hemodialysis

**DOI:** 10.3390/diagnostics15243123

**Published:** 2025-12-08

**Authors:** I-Min Su, Tsung-Jui Wu, Chin-Hung Liu, Yu-Li Lin, Bang-Gee Hsu

**Affiliations:** 1 Department of Anesthesiology, Dalin Tzu Chi Hospital, Buddhist Tzu Chi Medical Foundation, Chiayi 62247, Taiwan; 2 School of Medicine, Tzu Chi University, Hualien 97004, Taiwan; 3 Institute of Medical Sciences, Tzu Chi University, Hualien 97004, Taiwan; 4 Division of Nephrology, Department of Medicine, Hualien Armed Forces General Hospital, Hualien 97144, Taiwan; 5 Graduate Institute of Clinical Pharmacy, School of Medicine, Tzu Chi University, Hualien 97004, Taiwan; 6 School of Pharmacy, Tzu Chi University, Hualien 97004, Taiwan; 7 Division of Nephrology, Hualien Tzu Chi Hospital, Buddhist Tzu Chi Medical Foundation, Hualien 97004, Taiwan

**Keywords:** carotid–femoral pulse wave velocity, phenylacetylglutamine, hemodialysis, aortic stiffness

## Abstract

**Background/Objectives:** Phenylacetylglutamine (PAG), a gut microbiota-derived metabolite, may contribute to the development of cardiometabolic diseases. The diagnostic value of PAG for vascular dysfunction in hemodialysis (HD) remains unclear. This study assessed how serum PAG levels relate to carotid–femoral pulse wave velocity (cfPWV), which is a validated index of aortic stiffness, in patients on maintenance HD. **Methods:** A total of 138 patients on maintenance HD were enrolled. Participants with cfPWV values greater than 10 m/s were categorized as having aortic stiffness. Serum PAG levels were measured using high-performance liquid chromatography–mass spectrometry. **Results:** Aortic stiffness was present in 33.3% of patients. Those with aortic stiffness were older (*p* = 0.016), had a higher prevalence of diabetes mellitus (*p* = 0.030) and hypertension (*p* < 0.001), and had higher systolic (*p* < 0.001) and diastolic (*p* < 0.001) blood pressures, glucose (*p* = 0.005), and serum PAG (*p* < 0.001) levels. Multivariable analysis identified serum PAG (odds ratio [OR] = 1.903; 95% confidence interval [CI] = 1.171–3.094, *p* = 0.009) and age (OR = 1.042, 95% CI = 1.001–1.084, *p* = 0.044) as independent determinants of aortic stiffness. Linear regression revealed that PAG (*p* < 0.001), systolic blood pressure (*p* < 0.001), age (*p* = 0.013), and glucose level (*p* = 0.024) were positively associated with cfPWV. **Conclusions:** Among individuals undergoing maintenance HD, higher serum PAG levels independently predicted greater aortic stiffness. PAG may be a potential diagnostic biomarker for vascular stiffness and a tool for cardiovascular risk stratification in this population.

## 1. Introduction

Hemodialysis (HD) is the primary kidney replacement therapy for patients with end-stage renal disease (ESRD), accounting for approximately 90% of all dialysis treatments worldwide [1]. Among individuals receiving hemodialysis, cardiovascular disease represents the major contributor to overall mortality, with rates of up to 20-fold higher than those in the general population [2]. The excess cardiovascular (CV) risk reflects the interplay of both traditional and non-traditional risk factors, including hypertension, diabetes mellitus (DM), inflammation, oxidative stress, and the accumulation of gut-derived uremic toxins [3,4]. Notably, alterations in the gut–kidney axis have gained increasing attention as contributors to vascular dysfunction and CV disease (CVD) in ESRD [5,6].

Arterial stiffness, which is evaluated by pulse wave velocity (PWV), is an early and measurable manifestation of vascular injury and a strong predictor of CV morbidity and mortality in ESRD [7]. The carotid–femoral PWV (cfPWV) is widely recognized as the gold standard for evaluating large-artery elasticity and is strongly linked to CV outcomes in patients on HD [8,9]. Therefore, identifying novel biomarkers that can detect early vascular alterations could improve cardiovascular risk stratification in this population.

Phenylacetylglutamine (PAG) is a gut microbiota-derived metabolite that is generated from phenylalanine through microbial and hepatic conjugation pathways [6,10]. Elevated PAG levels are independently associated with significant adverse CV events, heart failure, and coronary artery disease [11,12,13]. PAG activates adrenergic receptors, resulting in increased platelet reactivity, endothelial dysfunction, and metabolic dysregulation [14,15]. These results suggest that PAG may function as a signaling molecule linking the gut microbiota to vascular pathologies.

Recent studies reported that PAG correlates with cfPWV in kidney transplant recipients [16] and predicts cardiovascular events in HD cohorts [17]. However, no study has investigated whether PAG is associated with aortic stiffness, as assessed by cfPWV, in patients undergoing maintenance HD. Patients on maintenance HD have a unique metabolic environment characterized by the accumulation of uremic toxins and gut microbial dysbiosis [18], which could enhance PAG-related vascular effects. Therefore, this study aimed to determine whether serum PAG concentrations are independently associated with aortic stiffness, as evaluated by cfPWV, in patients on maintenance HD. We further evaluated the diagnostic performance of PAG for detecting aortic stiffness to examine its potential clinical utility as a vascular biomarker in this high-risk population.

## 2. Materials and Methods

### 2.1. Patients

Overall, 138 patients on maintenance HD were enrolled from the dialysis unit of a clinical center in Hualien, Taiwan, from June to September 2022. Eligible patients were adults aged ≥20 years who had undergone 4 h HD sessions thrice weekly for at least 6 months. High-flux dialyzers made of polysulfone membrane from the FX series (Fresenius Medical Care, Bad Homburg, Germany) were routinely used during HD. Demographic data and medical history, including comorbidities such as DM and hypertension, were obtained from medical records. Ethical approval for this study was provided by the Research Ethics Committee of Hualien Tzu Chi Hospital, Buddhist Tzu Chi Medical Foundation (IRB108-219-A). All procedures were conducted in accordance with the ethical standards outlined in the Declaration of Helsinki. Patients were excluded if they declined participation, had a history of limb amputation, stroke, peripheral arterial occlusive disease, malignancy, liver cirrhosis, heart failure, had acute infection, or were bedridden.

### 2.2. Anthropometric and Biochemical Measurements

Measurements included height and body weight before and after the HD procedure. Body mass index was determined using the standard formula: weight (kg)/height (m^2^). Blood samples (5 mL) were collected prior to HD sessions on either Tuesday or Wednesday. Blood samples were collected using two separate tubes. For hemoglobin quantification, 0.5 mL of whole blood was drawn into an EDTA anticoagulant tube and analyzed using a Sysmex XS-1000i hematology analyzer (Sysmex America, Mundelein, IL, USA). For biochemical measurements, including serum PAG levels, an additional 4.5 mL of blood was collected in a serum separator tube without anticoagulant, allowed to clot, and then centrifuged at 3000× *g* for 10 min at 4 °C to obtain serum. Measurements of serum blood urea nitrogen, creatinine, albumin, total cholesterol, triglycerides, glucose, calcium, and phosphorus were performed with an automated clinical chemistry platform (Advia 1800; Siemens Healthcare GmbH, Henkestr, Erlangen, Germany). Dialysis adequacy was evaluated by calculating the single-pool fractional clearance index for urea (Kt/V) and the urea reduction ratio, which was based on pre- and post-HD urea levels, using a standard single-compartment kinetic model. A commercially supplied enzyme-linked immunosorbent assay (ELISA; Abcam, Cambridge, MA, USA) was used to measure serum intact parathyroid hormone (iPTH).

### 2.3. Blood Pressure and Aortic Stiffness Assessment

Brachial blood pressure was measured in a temperature-controlled and quiet setting with participants in the supine position following at least 10 min of rest. Systolic and diastolic blood pressures were measured three times at 5 min intervals using an automated oscillometric method, and the average values were used for analysis. cfPWV was quantified using a SphygmoCor XCEL volumetric cuff-based system (AtCor Medical; Sydney, NSW, Australia) [16]. Trained operators conducted all measurements in accordance with the manufacturer’s guidelines. The SphygmoCor XCEL system (XCEL 1.30 software) simultaneously and noninvasively records both carotid and femoral PWVs. The carotid pulse was captured using a high-fidelity tonometer placed gently over the carotid artery. Simultaneously, the femoral waveform was captured via a pneumatic cuff positioned around the upper thigh. Participants were instructed to remain still and refrain from talking during the procedures to minimize motion artifacts. Path length for cfPWV was derived using the subtraction approach, whereby the distance from the sternal notch to the femoral cuff is reduced by the distance from the carotid artery to the sternal notch. Using a flexible tape, distances were measured to the nearest millimeter. The system automatically calculated the cfPWV as the ratio of the estimated path length to the pulse transit time between the carotid and femoral sites, averaging over several consecutive cardiac cycles. Measurements with poor signal quality or excessive pulse-to-pulse variability were repeated until stable recordings were obtained. According to the European Society of Hypertension and European Society of Cardiology guidelines [19], patients with cfPWV values > 10 m/s were considered as having aortic stiffness. In contrast, those with cfPWV values ≤ 10 m/s were assigned to the control group.

### 2.4. Determination of Serum PAG Concentrations

HPLC–MS analysis was used to measure serum PAG, employing a Waters e2695 system coupled with an ACQUITY QDa mass detector (Waters Corporation, Milford, MA, USA) [20]. To ensure analytical precision, an isotopically labeled internal standard (d5-PAG, 25 ng/mL) was added. Briefly, 100 µL of serum and 400 µL of the internal standard diluent were combined in a 96-well plate, mixed, and incubated for 20 min. The plate was then centrifuged at 3000× *g* for 10 min at a temperature of 4 °C. A 150 µL aliquot of supernatant was then transferred to a new 96-well plate for further evaluation. Mass spectrometry was conducted in negative-ion mode with a full scan range of 100–350 *m*/*z*, monitoring PAG at 263.0 *m*/*z* and d5-PAG at 268 *m*/*z*, with retention times of approximately 9.8 min. Data acquisition and quantification were performed using the Empower 3.0 software (Waters, New York, NY, USA).

### 2.5. Statistical Analysis

Data normality was examined using the Kolmogorov–Smirnov test. Normally distributed variables are presented as mean ± standard deviation, and between-group comparisons were conducted using the two-tailed Student’s *t*-test. Skewed variables (HD duration, triglycerides, and iPTH) are presented as medians with interquartile ranges and compared using the Mann–Whitney U test. The non-normally distributed parameters were log_10_-transformed before further analyses. Categorical variables are expressed as numbers (percentages) and analyzed using the chi-square test. In the analysis comparing patients with and without aortic stiffness, variables with *p* < 0.2 were entered into a multivariable logistic regression model [21]. Relationships between cfPWV and clinical parameters were initially explored through univariable linear regression. Independent predictors were subsequently determined via multivariable forward stepwise linear regression. The capacity of serum PAG to differentiate patients with aortic stiffness was quantified using receiver operating characteristic (ROC) curve analysis, with area under the curve (AUC) values obtained in MedCalc (version 22.019, MedCalc Software Ltd., Ostend, Belgium). Statistical analyses were performed using Statistical Package for the Social Sciences (IBM Corp., Armonk, NY, USA), with a significance threshold of *p* < 0.05 (two-sided).

## 3. Results

### 3.1. Baseline Characteristics of the Study Population

Of the 138 patients on maintenance HD who were recruited, 46 (33.3%) and 92 patients had cfPWV values of >10 m/s and ≤10 m/s; they were classified into the aortic stiffness and control groups, respectively. The clinical and biochemical characteristics in both groups are presented in Table 1. Compared with the control group, patients with aortic stiffness were significantly older (*p* = 0.016) and had higher systolic (*p* < 0.001) and diastolic (*p* < 0.001) blood pressures. The prevalence of DM (*p* = 0.030) and hypertension (*p* < 0.001) was also significantly higher in the aortic stiffness group. Moreover, patients in this group had significantly higher glucose levels (*p* = 0.005) and serum PAG levels (*p* < 0.001). Other parameters, including dialysis adequacy (Kt/V), urea reduction ratio, and serum levels of lipid profile parameters, calcium, phosphorus, and iPTH, were not significantly different between the two groups.

### 3.2. Factors Associated with Aortic Stiffness

Variables with *p* < 0.2 in Table 1 were included in a multivariable logistic regression model (Table 2). After adjustment for age, DM, hypertension, systolic blood pressure, hemoglobin, albumin, phosphate, iPTH, and serum PAG—covariates that met the *p* < 0.2 criterion in univariable analysis—serum PAG remained independently associated with aortic stiffness (odds ratio [OR] = 1.903; 95% confidence interval [CI] = 1.171–3.094; *p* = 0.009). Additionally, age was also identified as an independent factor of aortic stiffness (OR = 1.042; 95% CI = 1.001–1.084; *p* = 0.044). Meanwhile, sex, dialysis duration, blood pressure, glucose level, DM, and hypertension were not significantly associated with aortic stiffness in the multivariable logistic regression model.

### 3.3. Determinants of cfPWV

Simple linear regression analysis showed that cfPWV values were associated with age (*r* = 0.275, *p* = 0.001), systolic blood pressure (*r* = 0.436, *p* < 0.001), diastolic blood pressure (*r* = 0.315, *p* < 0.001), glucose level (*r* = 0.236, *p* = 0.006), and serum PAG level (*r* = 0.446, *p* < 0.001) (Table 3). In the multivariable forward stepwise linear regression model, PAG concentration (β = 0.313, adjusted R^2^ change = 0.193, *p* < 0.001), systolic blood pressure (β = 0.292, adjusted R^2^ change = 0.095, *p* < 0.001), age (β = 0.180, adjusted R^2^ change = 0.025, *p* = 0.013), and glucose level (β = 0.162, adjusted R^2^ change = 0.019, *p* = 0.024) were independent predictors of cfPWV > 10 m/s. These findings indicate that serum PAG is a predictor of aortic stiffness.

### 3.4. Predictive Performance of Serum PAG

**Figure 1:**
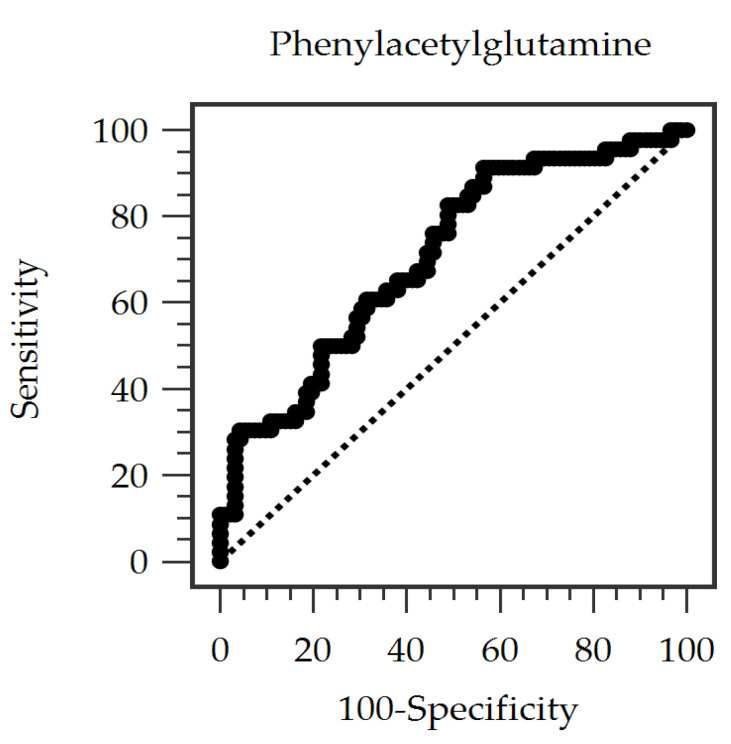
Receiver operating characteristic (ROC) curve analysis showing the predictive value of serum phenylacetylglutamine (PAG) levels for identifying aortic stiffness in 138 hemodialysis patients.

**Figure 2:**
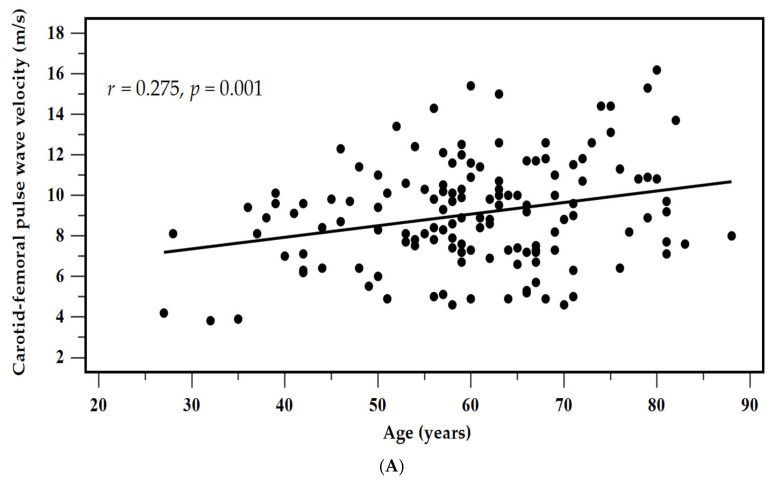

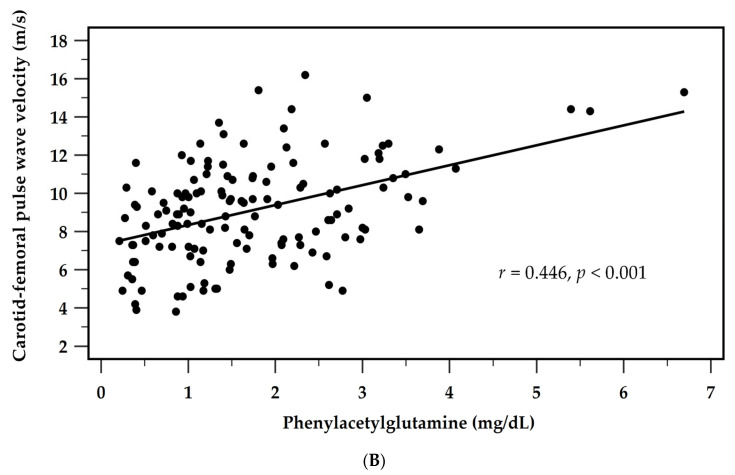
Scatter plots illustrating the relationships between cfPWV and (**A**) age and (**B**) serum PAG levels. These visual trends complement the statistical results presented in Table 3.

The diagnostic ability of serum PAG to identify aortic stiffness was then examined using ROC curve analysis (Figure 1). The AUC of serum PAG was 0.707 (95% CI = 0.616–0.798, *p* < 0.001), indicating a fair discriminative capacity. A cutoff PAG concentration of 1.026 mg/dL yielded a sensitivity of 91.3%, a specificity of 43.4%, a positive predictive value of 44.6%, and a negative predictive value of 90.9% for identifying aortic stiffness. Scatter plots with regression lines further revealed a positive linear relationship between cfPWV values > 10 m/s and both age and PAG concentrations (Figure 2A,B).

## 4. Discussion

This study fills an existing gap in the literature by identifying an independent association between elevated serum PAG levels and aortic stiffness in maintenance HD patients. Patients with aortic stiffness were older, more likely to have DM and hypertension, and had higher systolic and diastolic blood pressures as well as glucose and serum PAG concentrations. Following multivariable adjustments, PAG remained an independent predictor of both aortic stiffness and elevated cfPWV, suggesting that it may be a potential biomarker reflecting vascular injury beyond traditional risk factors. To our knowledge, the present study is the first to demonstrate an independent association between serum PAG levels and cfPWV—the gold standard measure of aortic stiffness—in patients undergoing maintenance HD. These results suggest that PAG may serve as a clinically relevant biomarker reflecting large-artery injury in this population.

Evaluations of baseline characteristics showed that patients with aortic stiffness were older and had higher systolic and diastolic blood pressures compared with those without stiffness, and that DM and hypertension, as well as elevated glucose levels, were more common in this group. These findings are aligned with the well-established influence of age, hypertension, and impaired glucose metabolism on arterial compliance. Aging causes elastin fragmentation and collagen accumulation within the arterial wall, thereby increasing vascular rigidity [22]. Similarly, chronic elevations in blood pressure cause mechanical stress that induces vascular remodeling and arterial stiffening [23]. Both hyperglycemia and diabetes facilitate the generation of advanced glycation end-products and are implicated in endothelial impairment and inflammation [24,25]. Collectively, these findings reinforce the current evidence that traditional CV risk factors are key determinants of aortic stiffness in the HD population, alongside emerging markers such as PAG.

PAG is a gut microbiota-derived metabolite of phenylalanine that has recently gained attention for its roles in cardiovascular and metabolic regulation [26]. Elevated PAG levels are associated with systemic inflammation, platelet activation, endothelial dysfunction, vascular calcification, and major adverse CV events [11,27,28,29]. In our maintenance HD cohort, higher serum PAG levels were independently associated with aortic stiffness, even after adjusting for conventional risk factors, including age, hypertension, DM, and glucose level. Stepwise linear regression further identified PAG, systolic blood pressure, age, and glucose levels as independent predictors of cfPWV > 10 m/s, collectively explaining a substantial proportion of the variability in aortic stiffness. These findings are aligned with those in kidney transplant recipients [16] in whom serum PAG levels were also independently correlated with cfPWV values and conventional CV risk factors. The convergence of results across different kidney disease populations reinforces the potential of PAG as a biomarker for vascular dysfunction and underscores its relevance across diverse clinical contexts.

Several processes may be attributed to the observed association between PAG and aortic stiffness. PAG can activate α- and β-adrenergic receptors, inducing oxidative stress and inflammation that impair endothelial function [6,15,30]. Additionally, other microbiota-derived metabolites, including p-cresyl sulfate and indoxyl sulfate, also induce vascular calcification and osteogenic transformation of vascular smooth muscle cells [31,32]. This suggests that PAG may contribute to vascular remodeling through related but unconfirmed mechanisms. Altogether, these mechanisms provide a plausible pathophysiological link between gut dysbiosis, elevated PAG levels, and increased vascular stiffness in patients on maintenance HD.

Clinically, our findings highlight PAG as a potential biomarker reflecting vascular dysfunction in patients undergoing maintenance HD. The ROC analysis showed that serum PAG moderately identified individuals with aortic stiffness (AUC = 0.707), demonstrating high sensitivity at the optimal cutoff value. This suggests that incorporating PAG into CV risk assessment may help detect early vascular injury before overt clinical events occur. Nonetheless, the specificity (43.4%) and positive predictive value (44.6%) were relatively low. This is not unexpected, as aortic stiffness in HD patients is driven by multiple coexisting factors—including aging, chronic inflammation, disordered mineral metabolism, and prolonged dialysis exposure—none of which can be fully captured by a single biochemical marker. Therefore, PAG should not be viewed as a stand-alone diagnostic test for aortic stiffness. Rather, it may serve as a complementary indicator of vascular injury or as a risk-stratification marker when integrated with established clinical and biochemical parameters. Future studies are warranted to explore whether combining PAG with other uremic toxins or vascular biomarkers could enhance predictive performance.

This study has several strengths, including the use of cfPWV and rigorous application of multivariable analyses. However, certain limitations should be noted. As the study is cross-sectional, it does not permit causal interpretation. Longitudinal follow-up studies are necessary to establish whether PAG forecasts future CV events. Furthermore, our cohort was relatively small and limited to a single center, which may restrict the generalizability of the results. Information on established CVD (e.g., coronary artery disease, myocardial infarction, heart failure, stroke) was not collected in this study, which limits our ability to evaluate the impact of pre-existing CVD on the association between PAG and aortic stiffness. The lack of data on diet, gut microbiota composition, and detailed medication profiles— including antiplatelet agents, statins, antihypertensive therapies, and antidiabetic drugs—as well as lifestyle factors, limits our ability to fully control for potential confounders, which may introduce bias into the association between PAG and aortic stiffness. Nevertheless, the consistent and independent association between PAG and cfPWV provides a compelling rationale for further mechanistic and interventional studies.

## 5. Conclusions

Elevated serum PAG levels are independently associated with increased aortic stiffness in patients on maintenance HD. PAG shows robust potential as a diagnostic biomarker for vascular stiffness and CV risk stratification. Future studies should investigate whether targeting gut-derived metabolites, through dietary modification, prebiotics, or pharmacological interventions, can attenuate aortic stiffness and improve CV outcomes in this vulnerable population.

## Figures and Tables

**Table 1:** Clinical and biochemical characteristics of 138 patients undergoing maintenance hemodialysis, stratified by the presence or absence of aortic stiffness (cfPWV > 10 m/s).

Characteristics	All Patients (*n* = 138)	Control Group (*n* = 92)	Aortic Stiffness Group (*n* = 46)	*p* Value
Age (years)	60.33 ± 12.30	58.57 ± 12.85	63.87 ± 10.34	0.016 *
Female, *n* (%)	68 (49.3)	50 (54.3)	18 (39.1)	0.092
Height (cm)	162.11 ± 9.43	161.40 ± 9.07	163.52 ± 10.08	0.215
Pre-HD body weight (kg)	68.67 ± 16.69	68.05 ± 16.98	69.91 ± 16.20	0.538
Post-HD body weight (kg)	66.45 ± 16.27	65.91 ± 16.64	67.52 ± 15.65	0.586
Body mass index (kg/m^2^)	25.97 ± 5.08	25.97 ± 5.35	25.97 ± 4.55	0.996
Systolic blood pressure (mmHg)	152.72 ± 28.35	144.76 ± 26.48	168.65 ± 25.31	<0.001 *
Diastolic blood pressure (mmHg)	80.17 ± 15.56	76.77 ± 13.59	86.98 ± 17.09	<0.001 *
Diabetes mellitus, % (*n*)	66 (47.8)	38 (41.3)	28 (60.9)	0.030 *
Hypertension, % (*n*)	83 (61.0)	44 (49.5)	38 (84.4)	<0.001 *
HD duration (months)	75.84 (33.300–144.00)	85.50 (45.57–144.93)	48.90 (27.36–138.18)	0.110
Urea reduction rate	0.72 ± 0.05	0.73 ± 0.05	0.72 ± 0.06	0.408
Kt/V (Gotch)	1.31 ± 0.19	1.31 ± 0.18	1.29 ± 0.21	0.488
Hemoglobin (g/dL)	10.70 ± 1.28	10.75 ± 1.33	10.59 ± 1.18	0.490
Albumin (g/dL)	4.35 ± 0.52	4.34 ± 0.52	4.37 ± 0.51	0.781
Total cholesterol (mg/dL)	148.53 ± 43.73	151.46 ± 45.35	142.67 ± 40.12	0.268
Triglyceride (mg/dL)	136.00 (90.50–202.50)	136.00 (91.25–203.50)	133.50 (86.50–189.50)	0.674
Glucose (mg/dL)	159.00 ± 63.70	148.45 ± 46.01	180.11 ± 85.97	0.005 *
Blood urea nitrogen (mg/dL)	65.21 ± 17.11	65.33 ± 16.95	64.98 ± 17.61	0.911
Creatinine (mg/dL)	10.16 ± 2.53	10.32 ± 2.57	9.84 ± 2.44	0.303
Total calcium (mg/dL)	9.35 ± 0.86	9.39 ± 0.90	9.27 ± 0.79	0.429
Phosphorus (mg/dL)	4.83 ± 1.40	4.93 ± 1.49	4.65 ± 1.18	0.268
iPTH (pg/mL)	288.55 (45.24–548.85)	269.55 (143.35–637.97)	290.95 (145.50–495.00)	0.957
Phenylacetylglutamine (mg/dL)	1.72 ± 1.13	1.44 ± 0.88	2.28 ± 1.35	<0.001 *
Carotid-femoral PWV (m/s)	9.09 ± 2.64	7.64 ± 1.67	12.00 ± 1.59	<0.001 *

Continuous variables are shown as mean ± SD or median (IQR), while categorical variables are given as *n* (%). Group comparisons used Student’s *t*-test, Mann–Whitney U test, or chi-square test, as appropriate. Abbreviations: HD, hemodialysis; PWV, pulse wave velocity; iPTH, intact parathyroid hormone; Kt/V, fractional clearance index for urea. Statistical significance: * *p* < 0.05.

**Table 2:** Multivariable logistic regression assessing clinical predictors of aortic stiffness in a cohort of 138 maintenance HD patients.

Variables	Odds Ratio	95% Confidence Interval	*p* Value
Phenylacetylglutamine, 1 mg/dL	1.903	1.171–3.094	0.009 *
Age, 1 year	1.042	1.001–1.084	0.044 *
Female	0.589	0.227–1.528	0.276
Hemodialysis duration, 1 month	1.000	0.994–1.006	0.983
Glucose, 1 mg/dL	1.008	0.999–1.016	0.057
Systolic blood pressure, 1 mmHg	1.015	0.988–1.043	0.274
Diastolic blood pressure, 1 mmHg	1.033	0.992–1.077	0.119
Diabetes mellitus, present	1.298	0.457–3.688	0.625
Hypertension, present	1.017	0.268–3.861	0.980

Variables with *p* < 0.2 in the analysis (Table 1) were entered into the model. * *p* < 0.05 was considered statistically significant.

**Table 3:** Linear regression analyses—both univariable and multivariable—of clinical variables related to cfPWV among 138 maintenance HD patients.

Variables	Carotid-Femoral PWV (m/s)				
	Simple Correlation		Multivariable Linear Regression		
	*r*	*p* Value	Beta	Adjusted R^2^ Change	*p* Value
Age (years)	0.275	0.001 *	0.180	0.025	0.013 *
Log-HD duration (months)	−0.144	0.093	–	–	–
Height (cm)	0.116	0.175	–	–	–
Pre-HD body weight (kg)	0.093	0.279	–	–	–
Post-HD body weight (kg)	0.087	0.309	–	–	–
Body mass index (Kg/m^2^)	0.042	0.629	–	–	–
Systolic blood pressure (mmHg)	0.436	<0.001 *	0.292	0.095	<0.001 *
Diastolic blood pressure (mmHg)	0.315	<0.001 *	–	–	–
Hemoglobin (g/dL)	−0.003	0.972	–	–	–
Albumin (g/dL)	0.038	0.655	–	–	–
Total cholesterol (mg/dL)	−0.104	0.224	–	–	–
Log-Triglyceride (mg/dL)	−0.081	0.345	–	–	–
Glucose (mg/dL)	0.236	0.006 *	0.162	0.019	0.024 *
Blood urea nitrogen (mg/dL)	−0.027	0.750	–	–	–
Creatinine (mg/dL)	−0.123	0.150	–	–	–
Total calcium (mg/dL)	−0.005	0.592	–	–	–
Phosphorus (mg/dL)	−0.037	0.665	–	–	–
Log-iPTH (pg/mL)	0.026	0.761	–	–	–
Phenylacetylglutamine (mg/dL)	0.446	<0.001 *	0.313	0.193	<0.001 *
Urea reduction rate	−0.046	0.592	–	–	–
Kt/V (Gotch)	−0.037	0.665	–	–	–

Variables with skewed distributions (HD duration, triglycerides, and iPTH) were log-transformed before analysis. Variables significantly correlated with cfPWV in simple regression were entered into the multivariable stepwise model. HD refers to hemodialysis; PWV, pulse wave velocity; iPTH, intact parathyroid hormone; and Kt/V, the fractional clearance index for urea. * Statistical significance was defined as *p* < 0.05.

## Data Availability

The data presented in this study are available on request from the corresponding author. The data are not publicly available due to privacy and ethical restrictions involving patient confidentiality.

## Abbreviations

The following abbreviations are used in this manuscript:

BMI	Body mass index
BUN	Blood urea nitrogen
cfPWV	Carotid–femoral pulse wave velocity
CV	Cardiovascular
CVD	Cardiovascular disease
DM	Diabetes mellitus
ELISA	Enzyme-linked immunosorbent assay
ESRD	End-stage renal disease
HD	Hemodialysis
HPLC–MS	High-performance liquid chromatography coupled with mass spectrometry
iPTH	Intact parathyroid hormone
PAG	Phenylacetylglutamine
PWV	Pulse wave velocity

## References

[1] Bello A.K., Okpechi I.G., Osman M.A., Cho Y., Htay H., Jha V., Wainstein M., Johnson D.W. (2022). Epidemiology of haemodialysis outcomes. Nat. Rev. Nephrol..

[2] Cozzolino M., Mangano M., Stucchi A., Ciceri P., Conte F., Galassi A. (2018). Cardiovascular disease in dialysis patients. Nephrol. Dial. Transplant..

[3] Ahmadmehrabi S., Tang W.H.W. (2018). Hemodialysis-induced cardiovascular disease. Semin. Dial..

[4] Zoccali C., Mallamaci F., Adamczak M., de Oliveira R.B., Massy Z.A., Sarafidis P., Agarwal R., Mark P.B., Kotanko P., Ferro C.J. (2023). Cardiovascular complications in chronic kidney disease: A review from the European Renal and Cardiovascular Medicine Working Group of the European Renal Association. Cardiovasc. Res..

[5] Jankowski J., Floege J., Fliser D., Böhm M., Marx N. (2021). Cardiovascular disease in chronic kidney disease: Pathophysiological insights and therapeutic options. Circulation.

[6] Chen M.C., Kuo C.H., Lin Y.L., Hsu B.G. (2025). Gut-derived uremic toxins and cardiovascular health in chronic kidney disease. Tzu Chi Med. J..

[7] Blacher J., Guerin A.P., Pannier B., Marchais S.J., London G.M. (2001). Arterial calcifications, arterial stiffness, and cardiovascular risk in end-stage renal disease. Hypertension.

[8] Korjian S., Daaboul Y., El-Ghoul B., Samad S., Salameh P., Dahdah G., Hariri E., Mansour A., Spielman K., Blacher J. (2016). Change in pulse wave velocity and short-term development of cardiovascular events in the hemodialysis population. J. Clin. Hypertens..

[9] Laurent S., Cockcroft J., Van Bortel L., Boutouyrie P., Giannattasio C., Hayoz D., Pannier B., Vlachopoulos C., Wilkinson I., Struijker-Boudier H. (2006). Expert consensus document on arterial stiffness: Methodological issues and clinical applications. Eur. Heart J..

[10] Gawałko M., Agbaedeng T.A., Saljic A., Müller D.N., Wilck N., Schnabel R., Penders J., Rienstra M., van Gelder I., Jespersen T. (2022). Gut microbiota, dysbiosis and atrial fibrillation. Arrhythmogenic mechanisms and potential clinical implications. Cardiovasc. Res..

[11] Nemet I., Saha P.P., Gupta N., Zhu W., Romano K.A., Skye S.M., Cajka T., Mohan M.L., Li L., Wu Y. (2020). A cardiovascular disease-linked gut microbial metabolite acts via adrenergic receptors. Cell.

[12] Zheng Y., Yu B., Alexander D., Manolio T.A., Aguilar D., Coresh J., Heiss G., Boerwinkle E., Nettleton J.A. (2013). Associations between metabolomic compounds and incident heart failure among African Americans: The ARIC Study. Am. J. Epidemiol..

[13] Ottosson F., Brunkwall L., Smith E., Orho-Melander M., Nilsson P.M., Fernandez C., Melander O. (2020). The gut microbiota-related metabolite phenylacetylglutamine associates with increased risk of incident coronary artery disease. J. Hypertens..

[14] Xu J., Cai M., Wang Z., Chen Q., Han X., Tian J., Jin S., Yan Z., Li Y., Lu B. (2023). Phenylacetylglutamine as a novel biomarker of type 2 diabetes with distal symmetric polyneuropathy by metabolomics. J. Endocrinol. Investig..

[15] Arefin S., Mudrovcic N., Hobson S., Pietrocola F., Ebert T., Ward L.J., Witasp A., Hernandez L., Wennberg L., Lundgren T. (2025). Early Vascular Aging in Chronic Kidney Disease: Focus on Microvascular Maintenance, Senescence Signature and Potential Therapeutics. Transl. Res..

[16] Yang H.-H., Chen Y.-C., Ho C.-C., Hsu B.-G. (2024). Serum Phenylacetylglutamine among Potential Risk Factors for Arterial Stiffness Measuring by Carotid–Femoral Pulse Wave Velocity in Patients with Kidney Transplantation. Toxins.

[17] Shafi T., Meyer T.W., Hostetter T.H., Melamed M.L., Parekh R.S., Hwang S., Banerjee T., Coresh J., Powe N.R. (2015). Free Levels of Selected Organic Solutes and Cardiovascular Morbidity and Mortality in Hemodialysis Patients: Results from the Retained Organic Solutes and Clinical Outcomes (ROSCO) Investigators. PLoS ONE.

[18] Lin T.Y., Wu P.H., Lin Y.T., Hung S.C. (2021). Gut dysbiosis and mortality in hemodialysis patients. npj Biofilms Microbiomes.

[19] Williams B., Mancia G., Spiering W., Agabiti Rosei E., Azizi M., Burnier M., Clement D.L., Coca A., de Simone G., Dominiczak A. (2018). 2018 ESC/ESH Guidelines for the management of arterial hypertension. Eur. Heart J..

[20] Huang P.Y., Huang C.S., Lin Y.L., Chen Y.H., Hung S.C., Tsai J.P., Hsu B.G. (2023). Positive association of serum galectin-3 with the development of aortic stiffness of patients on peritoneal dialysis. J. Clin. Med..

[21] Malhotra R.K. (2020). Errors in the use of multivariable logistic regression analysis: An empirical analysis. Indian J. Community Med..

[22] Xu X., Wang B., Ren C., Hu J., Greenberg D.A., Chen T., Xie L., Jin K. (2017). Age-related impairment of vascular structure and functions. Aging Dis..

[23] Humphrey J.D. (2021). Mechanisms of vascular remodeling in hypertension. Am. J. Hypertens..

[24] Rask-Madsen C., King G.L. (2013). Vascular complications of diabetes: Mechanisms of injury and protective factors. Cell Metab..

[25] Paneni F., Beckman J.A., Creager M.A., Cosentino F. (2013). Diabetes and vascular disease: Pathophysiology, clinical consequences, and medical therapy: Part I. Eur. Heart J..

[26] Nemet I., Li X.S., Haghikia A., Li L., Wilcox J., Romano K.A., Buffa J.A., Witkowski M., Demuth I., König M. (2023). Atlas of gut microbe-derived products from aromatic amino acids and risk of cardiovascular morbidity and mortality. Eur. Heart J..

[27] Fu H., Kong B., Zhu J., Huang H., Shuai W. (2023). Phenylacetylglutamine increases the susceptibility of ventricular arrhythmias in heart failure mice by exacerbated activation of the TLR4/AKT/mTOR signaling pathway. Int. Immunopharmacol..

[28] Lekawanvijit S., Kompa A.R., Wang B.H., Kelly D.J., Krum H. (2012). Cardiorenal syndrome: The emerging role of protein-bound uremic toxins. Circ. Res..

[29] Yu F., Li X., Feng X., Wei M., Luo Y., Zhao T., Xiao B., Xia J. (2021). Phenylacetylglutamine, a novel biomarker in acute ischemic stroke. Front. Cardiovasc. Med..

[30] Song Y., Wei H., Zhou Z., Wang H., Hang W., Wu J., Wang D.W. (2024). Gut microbiota-dependent phenylacetylglutamine in cardiovascular disease: Current knowledge and new insights. Front. Med..

[31] Curaj A., Vanholder R., Loscalzo J., Quach K., Wu Z., Jankowski V., Jankowski J. (2024). Cardiovascular consequences of uremic metabolites: An overview of the involved signaling pathways. Circ. Res..

[32] Yin L., Li X., Ghosh S., Xie C., Chen J., Huang H. (2021). Role of gut microbiota-derived metabolites on vascular calcification in CKD. J. Cell Mol. Med..

